# Large-Scale Person Re-Identification Based on Deep Hash Learning

**DOI:** 10.3390/e21050449

**Published:** 2019-04-30

**Authors:** Xian-Qin Ma, Chong-Chong Yu, Xiu-Xin Chen, Lan Zhou

**Affiliations:** Beijing Key Laboratory of Big Data Technology for Food Safety, Beijing Technology and Business University, Beijing 100048, China

**Keywords:** person re-identification, image analysis, hash layer, quantization loss, Hamming distance, cross-entropy loss

## Abstract

Person re-identification in the image processing domain has been a challenging research topic due to the influence of pedestrian posture, background, lighting, and other factors. In this paper, the method of harsh learning is applied in person re-identification, and we propose a person re-identification method based on deep hash learning. By improving the conventional method, the method proposed in this paper uses an easy-to-optimize shallow convolutional neural network to learn the inherent implicit relationship of the image and then extracts the deep features of the image. Then, a hash layer with three-step calculation is incorporated in the fully connected layer of the network. The hash function is learned and mapped into a hash code through the connection between the network layers. The generation of the hash code satisfies the requirements that minimize the error of the sum of quantization loss and Softmax regression cross-entropy loss, which achieve the end-to-end generation of hash code in the network. After obtaining the hash code through the network, the distance between the pedestrian image hash code to be retrieved and the pedestrian image hash code library is calculated to implement the person re-identification. Experiments conducted on multiple standard datasets show that our deep hashing network achieves the comparable performances and outperforms other hashing methods with large margins on Rank-1 and mAP value identification rates in pedestrian re-identification. Besides, our method is predominant in the efficiency of training and retrieval in contrast to other pedestrian re-identification algorithms.

## 1. Introduction

Pedestrian re-identification is the task of searching for and finding the same pedestrian captured by different cameras or the same camera at different times. In recent years, due to its extremely important applications in video monitoring, human–computer interaction, and other fields, this research has been a hot topic in the field of computer vision. However, affected by many factors, such as variable shooting angles, surrounding circumstances, and pedestrian behaviors, pedestrian re-identification still faces tremendous challenges.

Traditional pedestrian re-identification research [1,2] mostly uses the picture-concerned operator to extract feature information, such as global features, local features, pedestrian image color, texture, edge, shape, and other characteristics of information for later re-identification. In recent research on measurement learning methods for nearest neighbor classification, Weinberger and Saul [3] proposed the large margin nearest neighbor (LMNN) method, which sets the boundary for the target neighbor and punishment for the non-matching points of the intrusion boundary. To avoid the problems of over-fitting in LMNN, Davis et al. [4] proposed the information theoretic metric learning (ITML) method, which balances satisfying the given similarity constraint and ensures that the measure is close to the initial distance function.

In recent years, methods based on approximate nearest neighbors [5] to retrieve large-scale image datasets have been proposed. Among them, hash learning is a new approximate nearest neighbor re-identification method that represents an image as a string of fixed length and makes similar samples have similar binary coding [6], which has excellent performance in large-scale image re-identification. Image hash re-identification methods generally consist of image feature extraction to obtain an image feature code and hash mapping of that feature code.

In the traditional hash re-identification method, when extracting the low-dimensional compact features of an image, the image color, texture, shape, gradient, and other underlying feature information can be attained by manipulating the picture with the designed descriptor operator. When applied in complex scenarios, this method often fails to cover all cases well. The data-independent hash method [7], unsupervised hash learning method [8,9,10,11,12,13], and supervised learning algorithm [14,15] mainly use related feature operators to extract features before hashing to retrieve images.

The method based on deep learning can obtain useful features from the data with better generalization, semantic feature extraction, and strong expression ability. In 2009, using the non-linear expression of the deep learning model, Salakhutdinov and Hinton [16] first proposed the hash learning method based on a deep learning algorithm, of which the input feature remains artificial. In 2014, Xia el al. [17] proposed the convolutional neural network hashing (CNNH) model, which divides the hash learning into two stages. In the first stage, the image-paired similarity matrix based on LMNN is decomposed into binary vectors to complete the binary coding of image data and to be used as the training tag of the second stage. In the second stage, the binary coding of the convolutional neural network (CNN) model fitting image is constructed, and the performance of the model is improved by the classification loss function. However, the image representation learned in this method does not react to the update of binary hash code. In 2015, on the basis of weighting the Hamming distance, Zhang et al. [18] proposed the Deep Regularized Similarity Comparison Hashing (DRSCH). In 2016, Peng and Li [19] achieved hash code mapping by introducing the hash function into the final layer of the deep learning network. The output of the final layer in the network is the hash code. Meanwhile, classification loss is considered, but the number of categories in the dataset is too high, which leads to a significant increase in the dimension of the hash code.

Pedestrian re-identification is a branch of image re-identification, but some mature methods in image re-identification have not been applied in the field of pedestrian re-identification. Inspired by the deep hash learning algorithm, this paper proposes convolutional feature network person hash learning (CFNPHL) for pedestrian re-identification, which could achieve the learning of hashing code in pedestrian images. By calculating the distance between the hashing codes of pedestrian images, the re-identification of large-scale pedestrian image data is realized. The pedestrian hash learning algorithm of deep convolutional feature network proposed in this paper is different from other hash learning algorithms. The main contributions of this paper are as follows:

(1) The hash layer is applied to the fully connected layer in the proposed algorithm, which generates a hash code to learn the pedestrian images in an end-to-end approach. Additionally, for the integrity of the information, the quantized loss function is utilized in the procedure of generating the hash code in the hash layer.

(2) In order to make the hash code from the same pedestrian obtained by the network be the same or similar and that from a different pedestrian be of great discrepancy, the Softmax layer is adopted after the hash layer, which utilizes the Softmax cross-entropy loss to realize the higher constraint on the generated hash code of the network.

The proposed deep convolution network can extract feature information from a pedestrian image. Then, after utilizing the connection between fully connected layers to learn the hash function and mapping the feature information into the corresponding hash code, the network constructs a classifier layer for the pedestrian image. These efforts could make this network suitable for any huge pedestrian re-identification datasets, instead of limited datasets.

## 2. Related Work

In recent years, deep learning has made unprecedented achievements in various research fields. With stronger ability to extract image feature information, deep convolutional network replaces the traditional method of extracting pedestrian feature information by using manually designed image feature expression operators [20,21,22,23,24,25,26] in the research on pedestrian recognition. Among the existing pedestrian re-identification learning methods, they are mainly divided into three categories: Representation Learning-, Metric Learning-, and Generative Adversarial Networks (GAN)-based method.

The pedestrian re-identification method based on Representation Learning is a common method [26,27,28,29,30], which mainly uses the CNN to automatically extract image feature information according to task requirements after training, and then classifies or verifies pedestrians according to extracted feature information to achieve the purpose of re-recognition. Metric Learning is the most widely used method in the field of image retrieval. In the pedestrian re-identification, the similarity between the same pedestrian and different peers is mainly recognized. In the process of network training, the distance between the same pedestrian images (positive sample pairs) is minimized, while the distance between different pedestrian images (negative sample pairs) is maximized. A commonly used method of measuring learning loss consists of contrastive loss [31], triplet loss [32,33,34] and quadruplet loss [35]. The GAN-based method [36,37,38,39] is proposed to address the problem that the current dataset of pedestrian recognition is small. 

By generating new pedestrian images, the result of pedestrian recognition can be improved. The current pedestrian re-identification methods utilize images for pedestrian retrieval. However, in practice, the storage of each image needs to take up some space, and the retrieval of the image reduces the speed of recognition. Therefore, a commonly used hash retrieval method in the image retrieval filed was adopted in this paper. The length of hash code can be set manually. Usually, the number of hash codes will be much smaller than the size of the original picture, which will save a lot of storage space and also improve the speed of pedestrian recognition.

## 3. CFNPHL Methods 

**Figure 1:**
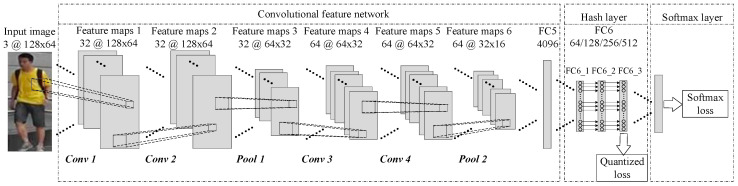
The network structure of the CFNPHL model.

The network structure of the CFNPHL model proposed in this paper is shown in Figure 1. The input data of this model are pedestrian images and label information. First, the convolutional feature network in the model is used to learn the feature information of pedestrian images. Then, the feature information of the pedestrian image is input into the full-connection layer of the network and the hash layer is introduced into the full-connection layer, so that the hash function can be learned and mapped into a hash code. Finally, in the process of training, the quantization loss of hash layer, and Softmax loss of classifier are updated to optimize network structure parameters.

As shown in Figure 1, the CFNPHL model we proposed is composed of three parts: convolutional feature network, hash layer and Softmax layer. In Figure 1, we take the 3-channel RGB color picture with a size of 128 × 64 as the input of the network for example. In the layer of convolution feature network, convolution and pooling are carried out, where con φ(φ=1,⋯,4) represents the convolution for four times and pool ε(ε=1,2) indicates max pooling for two times. Different feature maps could be obtained after operations. C@M×N indicates the size of the feature map is M × N with C dimension. The specific parameters and relevant processes of operation in this part will be introduced in detail in Section 3.1. The second part is the hash layer. The main purpose of this part is to convert the 4096-dimensional data of FC5 layer into the hash code of specific length, in which FC_1, FC_2, and FC_3 operate hash function mapping, Sigmoid function, and threshold, respectively. Moreover, it would be introduced in Section 3.2 specifically. The third part is the Softmax layer, which mainly differentiates the information of the same pedestrian and different pedestrians according to the category. This part is introduced in Section 3.3. 

### 3.1. Convolutional Feature Network

The function of the convolutional feature network is to learn the feature representation information of the pedestrian image. When the pedestrian image is input into the network, the image feature information of the pedestrian can be obtained through the convolutional feature network.

It is significant that the network could quickly extract the deep pedestrian feature information. Additionally, the network should be easy to be optimized. Therefore, the convolutional feature network in this paper adopts a structure of four CNN layers, two pooling layers and one full-connection layer, which is shown in Figure 1. The proposed hash layer does not constrain the network structure of convolution feature extraction, which means that the hash layer could be applied to other networks such as AlexNet or GoogLeNet. For example, if the input image size is 128 × 26, the parameter settings of the convolutional feature network are shown in Table 1. The dimension of pedestrian feature information extracted in the Pool2 layer of the convolutional feature network is relatively high. If it is directly connected with the FC5 of the full-connection layer, it can easily lead to over-fitting of the network. Thus, the dropout layer is introduced after the Pool2 layer, of which the purpose is to make the output of some nodes set to 0 with a certain probability in the training process. Pedestrian feature information obtained by the dropout layer is connected to the FC5 layer with a dimension of 4096, and the FC5 layer can obtain the representative information of pedestrian features extracted by the convolutional feature network.

The convolutional feature network in this paper has a shallow structural hierarchy. In the process of training, the network has a fast convergence speed and a short training time, and the network weight parameters are easily optimized. It can extract pedestrian image feature information with a strong expressive ability.

### 3.2. Hash Layer of the Network

Instead of the traditional algorithm to build the corresponding hash function mapping the feature information into the hash code, the mapping relationship between hash layers was introduced in this paper to represent the procedure of hashing code from the hash function. 

With the goals of efficient learning of the hash function and mapping into the hash code corresponding to the pedestrian image, this paper introduces a hash layer between FC5 and FC7 in the full-collection layer, of which the structure is depicted in Figure 1. By optimizing the weight of the network connection between FC5 of the full connection layer and FC6_1 of the hash layer, the pedestrian feature information used for FC5 layer learning $t_{i}(i=1,2,\cdots ,4096)$ is mapped into the hash function of the hash layer FC6_1 value $x_{j}(j=1,2,\cdots ,n)$. The activation function used in the convolutional feature network is the rectified linear unit (ReLU), which is defined below as Equation (1):

(1) f(x)=max(0,x)

The output of the hash layer ranges from [0,x) instead of a binary numeric string. Therefore, a constraint should be made to the hash layer FC6_1 so that it could generate a binary hash code. 

Then, the FC6_2 layer is introduced into the hash layer, which could use the Sigmoid function to activate the value from the FC6_1 layer so that the output range of the FC6_2 layer is [40,41]. The Sigmoid function is defined below as Equation (2):

(2) $$y=\frac{1}{1+e^{-x}}$$

where *y* denotes the output of the FC6_2 layer. Therefore, the value of FC6_2 is not a binary numeric string. To further obtain binary numeric strings, the FC6_3 layer is introduced into the hash layer, which quantizes the output of the FC6_2 layer. The quantized function is defined below as Equation (3):

(3) $$H(x)=\{\begin{array}{ll} 0, & y\leq K\\ 1, & y>K \end{array}$$

where *K* denotes a threshold. After Equation (3) is processed, the output of the FC6_3 layer is a binary hash code.

### 3.3. Loss Function of Network

In person re-identification, the deep networks often suffer from the over-fitting problem. It is difficult to solve the problem of overfitting by adjusting the hyperparameter of the network. In addition, changing the structure of the network can reduce the complexity of the network to a certain extent, but it is difficult to fundamentally solve the problem of overfitting in the problem of pedestrian re-identification. In this paper, in order to prevent the occurrence of network overfitting problem, we first design a shallow CNN on the network structure to extract the feature information of pedestrian images. Besides, two different loss functions are adopted to overcome the overfitting problem, namely using the measure loss function and the cross-entropy loss function to constrain the generated hash code value.

In the process of training the deep CNN, the weight parameters of the network need to be reversely adjusted by constantly reducing the error loss value of the network, of which the purpose is to achieve the optimal network. The error loss function in the CFNPHL model proposed in this paper consists of the following two parts.

#### 3.3.1. Quantized Loss Function of the FC6_3 layer in the Hash Layer. 

The quantized loss function of the FC6_3 layer in the hash layer indicates the information loss during the process that restricts the output of the FC6_3 layer to obtain the binary hash code.

For the purpose of minimizing the information loss, quantized loss is adopted, which is defined below as Equation (4):

(4) $$Loss_{q}=||y-H(x)|{|}_{2}^{2}$$

where *y* denotes the output of the FC6_2 layer and *H*(*x*) is mentioned in Equation (3). Motivated by quantized loss, the processed output of the FC6_2 layer in the hash layer will gradually approach 0 or 1 in the training process, which would result in less loss of information in the FC6_3 layer.

#### 3.3.2. The Softmax Cross-Entropy Loss Function

The Softmax classifier is used in the full-connection layer of the network to enable the model to learn the characteristic information that distinguishes different pedestrians, so that the characteristic codes of the same pedestrian are similar, and the characteristic codes of different pedestrians are not similar.

In the logical regression of the Softmax classifier, a tagged sample is used as the dataset: $\{(x^{\left(1\right)},y^{\left(1\right)}),$ $(x^{\left(2\right)},y^{\left(2\right)}),\cdots ,$ $\left(x^{\left(m\right)},y^{\left(m\right)})\right\}$, where $x^{\left(i\right)}\in R^{n+1}$ denotes a feature vector with n+1 dimensions. In the multi-classification, the value of y can be taken as k, where k is the number of classification categories, denoted as $y^{\left(i\right)}\in \{1,2,\cdots ,k\}$. The mission of the Softmax classifier is to approximate the probability that the input vector belongs to j. The function is defined below as Equation (5):

(5) $$h_{k}(x^{\left(i\right)})=\left[\begin{array}{c} p(y^{\left(i\right)}=1\left|x^{\left(i\right)};\theta_{1}\right|)\\ p(y^{\left(i\right)}=2\left|x^{\left(i\right)};\theta_{2}\right|)\\ \vdots \\ p(y^{\left(i\right)}=k\left|x^{\left(i\right)};\theta_{k}\right|) \end{array}\right]=\frac{1}{\sum_{j=1}^{k}e^{\theta_{j}^{T}x^{\left(i\right)}}}\left[\begin{array}{c} e^{\theta_{1}^{T}x^{\left(i\right)}}\\ e^{\theta_{2}^{T}x^{\left(i\right)}}\\ \vdots \\ e^{\theta_{k}^{T}x^{\left(i\right)}} \end{array}\right]$$

where $h_{k}(x^{\left(i\right)})$ indicates the output of the k^th^ Softmax layer based on the image of pedestrian $x^{\left(i\right)}\in R^{n+1}$, $\theta_{1},\theta_{2},\cdots ,\theta_{k}\in R^{n+1}$ denotes the parameters of the model, and T represents the number of iterations in network training. Besides, the final result of Equation (5) is the probability that the input $x^{\left(i\right)}\in R^{n+1}$ is classified into $y^{\left(i\right)}=j(k=1,2,\cdots ,k)$ category.

(6) $$Loss_{c}(\theta )=-\frac{1}{m}\left[\sum_{i=1}^{m}\sum_{j=1}^{k}1\{y^{\left(i\right)}=j\}\log\frac{e^{\theta_{j}^{T}x^{\left(i\right)}}}{\sum_{l=1}^{k}e^{\theta_{l}^{T}x^{\left(i\right)}}}\right]$$

where $Loss_{c}(\theta )$ denotes the cross entropy loss value, *m* is the number of training samples, *k* represents the number of pedestrian types and $1\{y^{\left(i\right)}=j\}$ is the characteristic function. If $y^{\left(i\right)}=j$ is true, the function is 1, otherwise not.

The total loss of CFNPHL is the sum of the quantized loss value and cross-entropy loss value. Consequently, the total loss is defined below as Equation (7):

(7) $$loss=Loss_{q}+Loss_{c}$$

where loss denotes the total loss, $Loss_{q}$ denotes the quantized loss mentioned in Equation (4), and $Loss_{c}$ denotes the Softmax cross-entropy loss mentioned in Equation (6). In the process of training, the network weight is reversely adjusted through the loss value so that the network gradually reaches the optimal value and loss gradually approaches zero.

### 3.4. Distance Re-Identification of Hash Code in a Pedestrian Image

In image re-identification, the Euclidean distance, Markov distance, Cosine distance, and Hamming distance are commonly used to measure image similarity. The pedestrian re-identification method used in this paper first converts the pedestrian image into the hash code, and then measures the hash code. In the information theory, the Hamming distance represents the number of different characters in the corresponding position of two equal-length strings. The Hamming distance is often considered to measure the equal-length hash codes generated in image retrieval methods [17,18]. In this paper, the hash code generated by the pedestrian image is used to measure the Hamming distance to realize pedestrian re-identification. The Hamming distance is expressed as $H(p_{x},p_{y})$, where $p_{x}$ denotes the hash code to be identified and $p_{y}$ denotes the generated pedestrian hash code library. 

**Figure 2:**
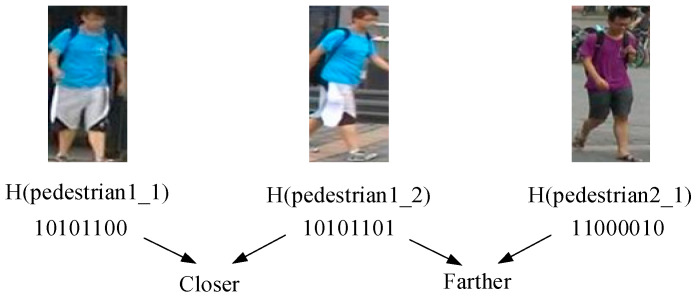
Hamming distance map.

The purpose of training CFNPHL model proposed in this paper is to make the Hamming distance of the hash code obtained by the same pedestrian closer and that of the hash code obtained by different pedestrians farther. A simple Hamming distance calculation example is provided in Figure 2 below, in which $p_{1,1}$ and $p_{1,2}$ are from the same pedestrian of which hash codes are 10,101,100 and 10,101,101, respectively, while $p_{2,1}$ is from a different pedestrian of which the hash code is 11,000,010. Supposing $p_{1,2}$ as the pedestrian image to be identified, the Hamming distance is calculated between the image to be identified and the image from the hash code library. Additionally, the result that $H(p_{1,2},p_{1,1})=1,H(p_{1,2},p_{2,1})=6$ could be obtained, which indicates the Hamming distance could measure the image similarity using the generated hash code. Eventually, the mission of pedestrian re-identification is completed by sorting the Hamming distance.

## 4. Experiments

The computer used in this experiment is configured as follows: Ubuntu16.04 + caffe 1.0 + Tensorflow1.2 + MATLAB 2016b; CPU: Intel i7 8700k; RAM: 16G ×2; GPU: NVIDIA GTX1080Ti.

### 4.1. Datasets

The experimental dataset consists of three kinds of pedestrian datasets: CUHK02 [40], Market-1501 [41], and DukeMTMC [42]. Brief descriptions of each dataset are as follows:

CUHK02 dataset [40]: A total of 7264 images of 1816 people were collected from 5 different outdoor cameras. Each person has four images, and the collected images are 160 × 60 in size. Two images of each person were taken at different times under each camera.

Market-1501 dataset [41]: This dataset was collected on the campus of Tsinghua University, and the images came from six different cameras, one of which was low-pixel. The image sizes are 64 × 128. Images were automatically detected and segmented by the detector, including some detection errors (close to the actual use). There are 751 people in the training data and 750 people are in the test set. Therefore, in the training set, each person has 17.2 pieces of training data on average.

DukeMTMC dataset [42]: The dataset was collected at Duke University, and images were taken from eight different cameras. This dataset provides a training set and a test set. The training set contains 16,522 images, the test set contains 17,661 images, and the query set contains 2228 images. There are 702 people in the training data, with an average of 23.5 pieces of training data for each person. The size of the image is not fixed, making it the largest pedestrian recognition dataset at present.

### 4.2. Settings

In this paper, two evaluation indexes are used to evaluate the performance of the hash method in the pedestrian re-identification dataset: 

(1) Mean average precision (mAP) [43]

(8) $$AP=\frac{1}{\sum_{i=1}^{m}r_{i}}\sum_{i=1}^{m}r_{i}(\frac{\sum_{j=1}^{i}r_{j}}{i})$$

(9) $$mAP=\frac{1}{M}\sum_{p=1}^{M}AP(p)$$

A pedestrian image is picked from the query set, and then m images with the highest similarity are selected from the candidate database. Therefore, in Equation (8), AP represents a probability that the query image is in the m images. If the image of pedestrian to be re-identified is consistent with the $i^{th}$ image in m images, $r_{i}$ is set 1, otherwise 0. In Equation (9), mAP is the mean of AP for M images in the query set.

(2) Cumulative match characteristic (CMC) [44]

(10) $$CMC(R)=\frac{1}{N}\sum_{q=1}^{N}\{\begin{array}{ll} 1, & r_{q}\leq R\\ 0, & r_{q}>R \end{array}$$

In Equation (10), with N pedestrians in the query set and N times of query and rank, the result of the target pedestrian in each query could be represented in $r=(r_{1},r_{2},\cdots ,r_{M})$.

In this paper, Rank-1, Rank-5, Rank-10 and Rank-20 are selected from the evaluation CMC metric. Among the CMC performance evaluation metrics, the Rank-1 is the most commonly used one.

To test and compare the advantages of the proposed method, the following three comparative experiments were carried out across the above four datasets.

(1) Contrast experiment with other hash algorithms

The hash algorithms compared in this paper include five unsupervised hashing methods, including Locality-Sensitive Hashing (LSH) [7], Spectral Hashing (SH) [9], Principal Component Analysis Hashing (PCAH) [10], Spherical Hashing (SPH) [12], Sparse Embedding and Least Variance Encoding (SELVE) [13] and three supervised hashing methods, including Binary Reconstructive Embeddings (BRE) [14], Minimal Loss Hashing (MLH) [15], and Principal Component Analysis directions and random matrix R (PCA-RR) [8]. The experimental results of these eight methods were obtained according to the parameter setting scheme provided by the corresponding authors.

(2) Influence of different hash code dimensions on CMC.

(3) Ignoring the quantized loss function proposed in this paper, we compared the CMC metric on various datasets.

(4) Pose invariant embedding (PIE) [26] versus the method proposed in this paper: The comparative experiment analysis on pedestrian re-identification was conducted on the dataset Market-1501. Besides, the relevant parameters of the experiment were set according to the parameters provided by the author.

For the above four comparative experiments, the settings of the three datasets used in this paper are as follows. In the CUHK02 dataset, of which the testing set contains 1816 images, one image of each person was randomly selected as the test dataset. For the unsupervised hash methods, the remaining images were considered as training sets. In the supervised hash method and the method proposed in this paper, two images were randomly selected from the unchosen/remaining images, with 3832 images as the training set and the rest as the test set.

For the dataset provided by Market-1501, each pedestrian image was randomly selected from the data as the testing set. In this paper, in the supervised hash method and the proposed method, the remaining images were used as training sets. In the unsupervised hash method, 80% of the remaining data were selected as the training set and the rest were chosen as the verification set.

For the DukeMTMC dataset, the image size was uniformly set to 200 × 80. In the supervised hash method and the method proposed in this paper, the training set, testing set and verification set were all provided by the original data. In the unsupervised hash method, the training set and testing set were unified as the training set, and the verification set remained unchanged.

The traditional hash algorithm involves two steps: extracting the characteristic information of the image by using the manually designed image analysis operator and generating the hash code from extracted feature information by establishing the corresponding hash learning algorithm. In the comparative experiment of traditional hash learning algorithm, instead of the traditional operator for feature extraction, it is the feature information of a 4096-dimension image extracted by pre-trained AlexNet as the input. Then, the hash algorithm of comparative experiment is utilized to generate the hash code. Eventually, the Hamming distance between hash codes is calculated for pedestrian re-identification.

### 4.3. Results and Analysis

#### 4.3.1. Comparison Across Diverse Hash Methods

**Figure 3:**
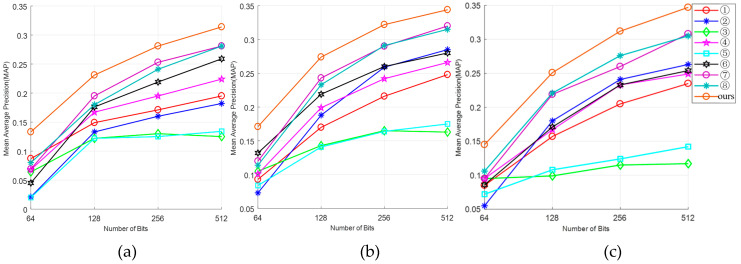
mAP values for different hash code dimensions of the three datasets: (**a**) mAP results of the CUHK02 dataset; (**b**) mAP results of the Market-1501 dataset; (**c**) mAP results of the DukeMTMC dataset.

The result shown in Figure 3 is the mAP value on three data points under the four kinds of hash code dimensions of 64 bits, 128 bits, 256 bits, and 512 bits. When the dimension of the hash code is 256 bits, Table 2 shows Rank-1 and mAP across the three datasets. 

As shown in Figure 3, eight sets of comparative experiments are indicated by the numbers ①–⑧, which represent LSH [7], SH [9], PCAH [10], SPH [12], SELVE [13], BRE [14], MLH [15], and PCA-RR [8], respectively. Additionally, ‘ours’ represents the proposed method.

It can be inferred from Figure 3 that our experimental results are significantly better than other comparative experimental methods. In the eight-group comparative experiment on pedestrian re-identification, the experimental results of MLH [15] and PCA-RR [8] with a supervised hashing learning method are better than those with an unsupervised hashing learning method. In addition, the experimental results of PCAH [10] and SELVE [13] are poor, and there is no significant improvement with the ascent of hash code dimension.

The following points can be gathered from the results:

1) Compared with other hash re-identification algorithms and the traditional hash algorithm (PCA-RR), the proposed method is equipped with a higher mAP value when pedestrian re-identification is performed across the three datasets. In addition, when the dimension of the hash code comes to 256 bits, the mAP values increase by 4%, 3.1%, and 3.5% for the respective datasets.

2) The mAP and Rank-1 are important evaluation metrics in the field of pedestrian re-identification. Conditioning on 256 bits of the hash code, Table 2 shows the result of both evaluations on three datasets. As can be inferred in Table 2, our method performs better than the traditional hash method in perspective of both evaluation metrics. It proves that our method can better learn the corresponding hash function according to the characteristics of the dataset and map it into the hash cod, which could improve the accuracy of pedestrian re-identification.

As can be seen from Figure 3 and Table 2, the mAP values and Rank-1 values obtained by PCAH and SELVE methods on the three datasets are relatively low. We hypothesize that a great deal of pedestrian feature information is lost when the algorithm processes the high-dimensional data, which leads to the hash code from the same pedestrian differing in similarity resulting in a low mAP value. It also could be seen from the result that supervised hash learning algorithms, MLH and PCA-RR, have greater mAP values than unsupervised learning algorithms, PCAH and SELVE. Besides, the results of the other algorithms like LSH, SH, SPH, and BRE are somewhat different. Our deep hashing network achieves the comparable performances and outperforms other hashing methods with large margins on Rank-1 and mAP value identification rates.

#### 4.3.2. Influence of Variant Dimension of Hash Code on CMC and mAP 

The effects of different dimensions of the hash layer on the CMC value and mAP is shown in Table 3 below.

As can be seen from Table 3, accompanied with the growth of the hash code’s dimension, the CMC metric and mAP increase simultaneously, in which the Rank-1 metric is significantly growing, and the Rank-10 and Rank-20 metrics are in a smooth growth when the dimension of hash code comes to 256 or 512 bits. The reason why such a phenomenon occurs is that the description of the feature is more detailed and concrete as the dimension of the hash code increases, which means that the probability of finding the same target in the first search is higher. Nevertheless, as the hash code length increases exponentially, the re-identification speed of the hard disk storage space rises. 

#### 4.3.3. Ignorance of the Quantized Loss Function Proposed 

To further investigate the influence of the quantized loss function on the result, the CMC metric and mAP, a comparative experiment was conducted to identify whether the quantized loss function was considered or not. Quantized loss was not considered in the training, only classification loss was used for model training, and the other experimental parameters were set in accordance with the settings considering quantized loss, in which the dimension of hash code was set to 256.

From the experimental results in Table 4, it can be concluded that across the three datasets, the Rank-1 metric values in CMC obtained by the model without considering the loss of measurement are reduced by 5.1%, 4.4%, and 4.2%, respectively. The loss measurement function proposed in this paper improves the re-identification accuracy of the model to some extent.

#### 4.3.4. Comparative Experiment Analysis of Pedestrian Re-Identification on the PIE Method

For the fact that Market-1501 is the common dataset between our method and PIE method [26], the comparative experiment and analysis were conducted merely on the dataset Market-1501.

There are many research methods for pedestrian re-identification at present, and the reason why we choose the PIE method could be concluded as follow. First, the PIE method is one of the classical and convincing methods to solve the problem of pedestrian re-identification. Second, the result of PIE method is satisfying, indicating that it was a dominant baseline in the pedestrian re-identification filed. The direct comparison experiment between PIE and ours can highlight the advantages of the proposed method. Therefore, we believe that comparison experiments using the PIE method can emphasize the contribution of this paper to some extent.

In this part of the experiment, the dimension of the hash layer was set as 512. The mAP values of the two pedestrian re-identification methods obtained through the experiment in the Market-1501 dataset and the results of Rank-1 in the CMC are shown in the Table 5.

It can be seen from the experimental results in Table 5 that the mAP value and Rank-1 value of our deep hash pedestrian re-identification algorithm are lower than the experimental results of PIE. The reason for such a situation could be concluded that in the procedure of mapping the pedestrian feature information into the hash code in the hash layer, the feature information of pedestrian gets compressed, which results in inevitable loss of feature information. While the PIE method is to recognize the feature information generated by deep CNN directly. Therefore, the PIE method performs better than our method in pedestrian re-identification.

In the network, the extracted pedestrian feature information changes from a high-dimensional to a low-dimensional hash code, which will inevitably cause the loss of pedestrian feature information. Therefore, the ability to maintain the important feature information is the key to improving the result of pedestrian re-identification after the transformation. As can be seen from Table 3, the use of hash codes with higher dimensions can improve the result of pedestrian re-identification to some extent, but the result rate of pedestrian recognition with higher dimensions becomes slower and the retrieval time becomes longer. Therefore, it is necessary to determine appropriate dimensions and improve the network’s ability to map pedestrian feature information into the hash code by using the hash function. In this way, excessive pedestrian feature information can be maintained and prevented from being lost, so as to maximize the efficiency of pedestrian re-identification with the hash code. 

**Figure 4:**
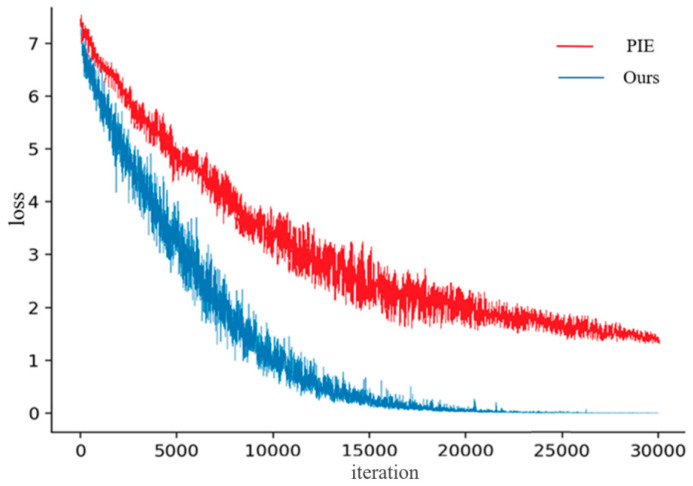
The loss value of CFNPHL and PIE.

However, during the experiment, the loss of our method was calculated by quantization loss and cross-entropy loss (Equation (7)). However, the loss of PIE method is the sum of Ori.img Softmax loss, PIE Softmax loss, and PoseBox Softmax loss. It was found that the convergence speed of loss values in the proposed CFNPHL model is faster than that of PIE, as shown in Figure 4.

It can be seen from Figure 4 that loss of CFNPHL model converges rapidly, and after 23,000 iterations, the loss value is close to 0. Compared with the CFNPHL, the PIE does not converge until 30,000 iterations are completed but the loss value is not close to 0.

**Figure 5:**
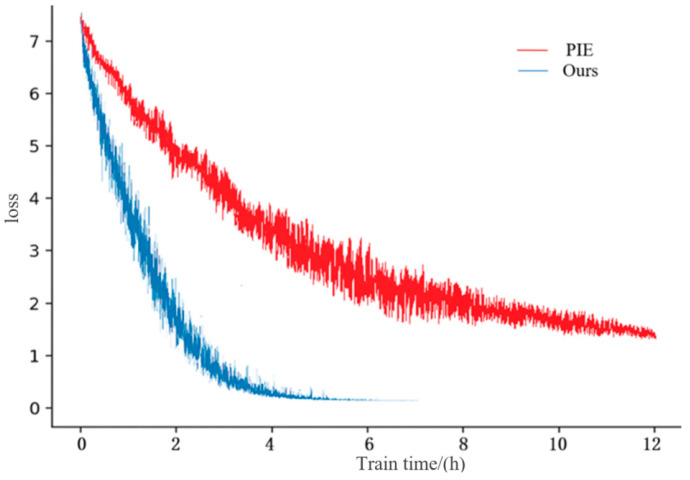
The train time costs of 30,000 iterations for CFNPHL and PIE.

Besides, as shown in Figure 5, the comparison of train time costs of 30,000 iterations in the same circumstance for both the models is conducted. PIE takes 12 h to converge while our proposed CFNPHL merely takes 7 h to converge. It could be inferred that the CFNPHL is trained more efficiently.

With the Market-1501 dataset for validation, our proposed method exceeds the PIE method in the speed of pedestrian re-identification. Moreover, the specific test time cost is shown in Table 6. Due to the transformation of pedestrian feature information extracted by the deep convolution network into the fixed length hash code, the dimension of data is immensely reduced. Therefore, our method achieves better performance than the PIE method.

As can be seen in Table 6, the PIE model spends much more time in testing than the CFNPHL model proposed in this paper. It can also be seen from the time taken by the different hash code dimensions of our model that the higher the hash code dimension, the longer the time taken.

## 5. Conclusions

Equipped with the deep neural network that has an excellent capacity to extract features and the hash layer that maps the pedestrian feature into a binary hash code, the CFNPHL model proposed in this paper utilizes quantized loss and the Softmax cross-entropy loss to achieve end-to-end training. This method applies the hash algorithm to the pedestrian re-identification domain for research, and results show that the proposed deep hash re-identification algorithm has a higher re-identification accuracy than other hash pedestrian re-identification algorithms. The experimental results show that compared with the PIE method, our method performs better in training time, convergence speed and retrieval time, but in the pedestrian recognition results (including Rank-1 and mAP), our method still needs to improve. Therefore, numerous endeavors could be devoted to researching hash algorithms in pedestrian re-identification.

## Figures and Tables

**Table 1:** Convolutional feature network parameter setting.

Layer	Output Size	Parameter Setting
Conv1	128 × 64 × 32	3 × 3, 32, pad = 0
Conv2	128 × 64 × 32	3 × 3, 32, pad = 0
Pool1	64 × 32 × 32	2 × 2, max pool, stride = 2
Conv3	64 × 32 × 64	3 × 3, 64, pad = 0
Conv4	64 × 32 × 64	3 × 3, 64, pad = 0
Pool2	32 × 16 × 64	2 × 2, max pool, stride = 2
FC5	4096	4096

**Table 2:** Comparative evaluation.

Method	CUHK02 [40]		Market-1501 [41]		DukeMTMC [42]	
	Rank-1	mAP	Rank-1	mAP	Rank-1	mAP
**LSH [7]**	20.3	17.1	23.5	21.6	23.3	20.5
**PCA-RR [8]**	26.8	24.1	34.3	29.5	29.5	27.6
**SH [9]**	18.1	16	28.6	25.9	27.9	24.1
**PCAH [10]**	14.3	13	18.9	16.5	15.4	11.5
**SPH [12]**	21.4	19.5	28.4	24.2	26.3	23.3
**SELVE [13]**	15.7	12.5	19.2	16.4	16.1	12.4
**BRE [14]**	23.4	21.9	30.4	26.1	25.4	23.4
**MLH [15]**	27.7	25.3	35.1	29.3	28.8	26.0
**Our**	**29.3**	**27.5**	**38.1**	**34.4**	**31.5**	**30.2**

**Table 3:** Effects of hash codes of different dimensions on CMC values and mAP.

Dataset	Hash Code Dimension	CMC				mAP
		Rank-1	Rank-5	Rank-10	Rank-20	
**CUHK02 [40]**	**64**	14.2	18.1	25.4	31.9	13.3
	**128**	24.1	31.3	39.2	46.7	22.1
	**256**	29.3	34.4	44.3	51.8	27.5
	**512**	**33.1**	**40.2**	**47.4**	**57.2**	**31.4**
**Market-1501 [41]**	**64**	17.4	24.2	29.6	40.1	17.1
	**128**	27.2	31.1	36.4	47	25.9
	**256**	33.1	35.4	45.9	56.3	31.2
	**512**	**38.1**	**46.3**	**55.2**	**65.8**	**34.4**
**DukeMTMC [42]**	**64**	15.1	17.2	22.3	28.3	14.5
	**128**	25.5	30.3	39.1	42.5	24.1
	**256**	31.5	38.1	40.5	46.1	30.2
	**512**	**36.5**	**40.5**	**47.8**	**54.9**	**33.5**

**Table 4:** Effects of quantitative loss function on CMC values of experimental results.

Dataset	Method	CMC				mAP
		Rank-1	Rank-5	Rank-10	Rank-20	
**CUHK02 [40]**	Our-	24.2	32.8	41.9	49.3	20.2
	**Our**	**29.3**	**34.4**	**44.3**	**51.8**	**27.5**
**Market-1501 [41]**	Our-	28.7	31.0	40.8	53.2	25.4
	**Our**	**33.1**	**35.4**	**45.9**	**56.3**	**31.2**
**DukeMTMC [42]**	Our-	27.3	34.9	0.371	0.413	24.3
	**Our**	**31.5**	**38.1**	**40.5**	**46.1**	**30.2**

**Table 5:** The mAP and Rank-1 on the Market-1501 [41] dataset.

Method	Rank-1	mAP
**PIE [26]**	**78.65**	**53.87**
**Ours**	38.1	34.4

**Table 6:** Test time of CFNPHL and PIE on the Market-1501 dataset.

Method	Test Time (min)			
**PIE [26]**	25.3			
**Ours**	**64 bits**	**128 bits**	**256 bits**	**512 bits**
	5.4	7.1	11.5	17.7
